# Genome classification by gene distribution: An overlapping subspace clustering approach

**DOI:** 10.1186/1471-2148-8-116

**Published:** 2008-04-23

**Authors:** Jason Li, Saman K Halgamuge, Sen-Lin Tang

**Affiliations:** 1 Bioinformatics Section, Biomechanical Engineering, Department of Mechanical Engineering, the University of Melbourne, Australia; 2 Research Center for Biodiversity, Academia Sinica, Taiwan

## Abstract

**Background:**

Genomes of lower organisms have been observed with a large amount of horizontal gene transfers, which cause difficulties in their evolutionary study. Bacteriophage genomes are a typical example. One recent approach that addresses this problem is the unsupervised clustering of genomes based on gene order and genome position, which helps to reveal species relationships that may not be apparent from traditional phylogenetic methods.

**Results:**

We propose the use of an overlapping subspace clustering algorithm for such genome classification problems. The advantage of subspace clustering over traditional clustering is that it can associate clusters with gene arrangement patterns, preserving genomic information in the clusters produced. Additionally, overlapping capability is desirable for the discovery of multiple conserved patterns within a single genome, such as those acquired from different species via horizontal gene transfers. The proposed method involves a novel strategy to vectorize genomes based on their gene distribution. A number of existing subspace clustering and biclustering algorithms were evaluated to identify the best framework upon which to develop our algorithm; we extended a generic subspace clustering algorithm called HARP to incorporate overlapping capability. The proposed algorithm was assessed and applied on bacteriophage genomes. The phage grouping results are consistent overall with the Phage Proteomic Tree and showed common genomic characteristics among the TP901-like, Sfi21-like and sk1-like phage groups. Among 441 phage genomes, we identified four significantly conserved distribution patterns structured by the terminase, portal, integrase, holin and lysin genes. We also observed a subgroup of Sfi21-like phages comprising a distinctive divergent genome organization and identified nine new phage members to the Sfi21-like genus: *Staphylococcus *71, phiPVL108, *Listeria *A118, 2389, *Lactobacillus phi *AT3, A2, *Clostridium *phi3626, *Geobacillus *GBSV1, and *Listeria monocytogenes *PSA.

**Conclusion:**

The method described in this paper can assist evolutionary study through objectively classifying genomes based on their resemblance in gene order, gene content and gene positions. The method is suitable for application to genomes with high genetic exchange and various conserved gene arrangement, as demonstrated through our application on phages.

## Background

One of the key problems in computational biology is the detection of evolutionary relationships using genomic information. For higher organisms, such relationships are often computed as a phylogenetic tree according to criteria such as the divergence of primary sequences, gene content, and gene order [1]. For microorganisms including viruses and bacteriophages, however, a phylogenetic tree may not completely describe their relationship because of the relatively large amount of horizontal gene transfers (HGT) in their evolutionary history [2-4]. Consequently, alternative strategies such as genome classification based on gene distribution [5] and classification based on short nucleotide sequences [6] have recently been proposed to provide different perspectives for understanding their genomic relationships. These strategies may not independently provide a complete description of evolutionary history, but they undoubtedly offer evolutionary insights that may not be obtained from tree-based phylogeny.

**Figure 1:**
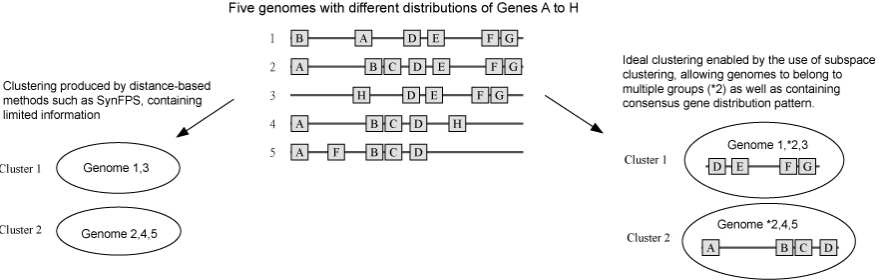
Illustration of the genome classification problem and additional information enabled by subspace clustering.

Gene-distribution-based classification or clustering refers to the task of identifying and grouping genomes with similar gene content, gene order, and positional coupling within local or global genomic segments (the concept of "local" and "global" here is analogous to that in sequence alignment). Although a number of computational methods related to gene distribution and genome rearrangement are currently available, these methods focus mainly on the close inspection of a few related species and tree reconstructions, and are not capable of discovering clusters among a large collection of genomes. Details of these methods are provided in the Discussion section. The pioneering method that is capable of clustering and providing evolutionary insights for a large number of genomes including distant species was proposed only recently [5]. The method, SynFPS, derives a score for each pair of genomes from gene-gene distances and then applies K-means over the pairwise scores to produce genome clustering [5]. The method has two major limitations. Firstly, although genome clusters are derived from gene distribution, the algorithm cannot dictate the consensus gene distribution pattern of each cluster. Knowing what species are related but not knowing the exact basis on which they are related can hinder further investigation of species relationships. Secondly, each genome is clustered into exactly one group, preventing a species from belonging to multiple clusters (overlapping clustering). This prohibits analysis of the genomes within which multiple conserved gene arrangement patterns have been acquired through HGT. The clustering problem itself and these two limitations are illustrated in Figure 1.

Motivated by the need for overlapping clustering and consensus gene pattern identification, we propose in this paper an overlapping subspace clustering technique for genome classification. Subspace clustering, also known as projected clustering and biclustering, is aimed at identifying objects that are similar in subspaces of the input space (the object space) [7,8]. If a dataset contains *M *data and *N *dimensions, traditional clustering would identify one or more clusters within the dataset, with each cluster containing *m *≤ *M *data that are similar in the ℜ^*N *^space. In contrast, subspace clustering would further associate each cluster with a subset of dimensions, such that each cluster would contain data that are only similar in its associated subspace *S *⊂ ℜ^*N*^. Subspace clustering can be further classified as disjoint or overlapping. In disjoint clustering, each object can only be assigned to one or no (outlier) clusters, whereas in overlapping clustering, each object can be assigned to any number of clusters.

Our research began with the creation of an evaluation data set that models the difficult issues often encountered in genome clustering problems (reported in Methods). A number of recent and popular subspace clustering algorithms were then evaluated for their performance on the evaluation data set. As not all these algorithms are capable of producing overlapping clustering, a number of different evaluation measures were employed. We then modified the best performing algorithm, HARP, to achieve enhanced accuracy as well as overlapping capability. The modified algorithm that we propose is called O-HARP [9]. Clustering results generated by O-HARP can assist evolutionary study by objectively classifying the genomes based on their resemblance in gene order, gene content and genome positions. The algorithm is suitable for application to genomes with high genetic exchange and various conserved gene arrangements. Bacteriophage (phage) genomes are an example and are the application focus of this work. Phages are particularly suitable for gene-distribution-based clustering analysis because they have undergone extensive HGT while their genomes still preserve certain conservations of gene order and gene position [10]; extensive HGT events have caused an inadequacy of the present phage taxonomic classification system [11] and thereby clustering based on conserved gene arrangement can provide augmented evolutionary insights.

## Results

### Method overview

**Figure 2:**
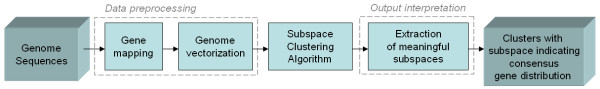
The overall process of detecting subspace clusters from a collection of genome sequences.

The application of subspace clustering to genome classification requires data preprocessing and output interpretation. The components involved in this process are illustrated in Figure 2.

*Gene mapping *refers to the determination of gene-gene correspondence across the genome sequences. The objective of this step is to define a group of genes which distribution will be used as the basis for genome classification. For our experiments where phages are the focus, we detect gene correspondence in two steps. Firstly, BLASTP with Blosum62 was used to group together genes with significant sequence similarity (E-value < 0.1) [12]. In the second step, genome annotation mining based on regular expression [5] was employed to bring analogous gene groups together, targeting the problem of divergent phage genes [5]. Each resulting group consists of a set of analogous genes, hereafter all treated equally; protein distance information is discarded after gene grouping.

*Genome vectorization *refers to the representation of gene distribution information as numeric vectors. We propose that each genome be represented by two pieces of information: the relative genome positions between all possible pairs of genes and the absolute positions of the genes. For example, let there be a genome G1 with gene A located at position 10 bp (10 basepairs from the start of the genome), gene B at 60 bp, and gene C at 30 bp. Let there be another genome G2 with gene A at 15 bp, gene B at 50 bp and gene C absent. The numeric vectors of these two genomes are shown in Table 1. The values for relative positions are signed (e.g. negative value for dimension "B-C" in G1), thus capturing gene order information as well as gene-gene distances. A gene may be present in one genome while being absent in another. In this case, the values associated with the missing gene in a genome will not be available, and can be implemented as NaN (not a number) in many modern computing languages such as Java. These missing values can be naturally handled by axis-aligned subspace clustering algorithms, as one of their fundamental abilities is to filter out subsets of dimensions. With this vectorization technique, the use of *n *genes will lead to a total of *nC*2+*n *dimensions.

**Table 1:** Example of genome vectorization.

	A-B	A-C	B-C	A	B	C
G1	50	20	-30	10	60	30
G2	45	n/a	n/a	15	50	n/a

The first three dimensions capture gene order and gene- gene distance information. The last three dimensions capture positional information.

A *subspace clustering algorithm *(e.g. O-HARP) processes the vectorized gene distribution data and produces a set of clusters as the output. Each cluster contains a set of genomes and is associated with a subspace that dictates the common gene distribution pattern of that cluster.

*Extraction of meaningful subspaces *is a procedure to remove clusters that have subspaces corresponding to a *non-continuous *gene distribution. A *continuous *distribution is defined to represent a conserved pattern among all the genes of interest; unless conserved property is observed in each pair of genes, the distribution is regarded as *non-continuous*. Enforcing continuous gene distribution can reduce the size and enhance interpretability of the resulted clusters.

### The proposed overlapping subspace clustering algorithm: extension to HARP

We evaluated a number of recent and popular subspace clustering algorithms (see next subsection) and identified HARP [13] as the best existing algorithm in terms of subspace clustering accuracy (SCE), clusters coverage (CI) and correct number of clusters (DNC) (refer to Methods: Performance measures). HARP is a relatively recent algorithm designed for general subspace clustering and has been reported with performance superior to PROCLUS [14], ORCLUS [15] and FastDOC [16]. HARP uses an agglomerative hierarchical approach, in which the algorithm begins by considering each individual data as a separate cluster and subsequently builds up larger clusters by merging the smaller ones. With such an approach, at least one pair of clusters should be merged in every iteration of the algorithm, and therefore a criterion is needed to decide which pair is to be merged next. To achieve this, HARP uses a *merge score *to rate how well two clusters can be merged. If there is a total of *n *clusters, then there are *nC*2 (*n *choose 2) merge scores that need to be computed. To reduce computational complexity, the authors proposed the use of individual statistics (e.g. means and variances) of the *n *separate clusters to compute the merge scores instead of using statistics of the *nC*2 potential clusters. Nevertheless, such merge score have been developed for disjoint clustering only and bias exists in the merge of unequal-sized clusters.

In this work, we propose a few modifications to HARP to enable overlapping clustering as well as to improve performance for gene-distribution-based genome clustering. The resulting algorithm is called O-HARP.

#### A) Merge score

We propose the following merge score to handle overlapping clusters and to improve general clustering performance. Suppose we have a cluster denoted as *C*_*i*_. For each dimension *j*, a *local variance *$\sigma_{ij}^{2}$ is computed as the variance across all the *j*^*th *^dimensional values of the data within *C*_*i*_, and a *local mean μ*_*ij *_is defined similarly. Also, a *global variance *$\sigma_{j^{\prime }}^{2}$ is computed as the variance across all values within the dataset *that are associated *with the *j*^*th *^dimension. Such association depends on the problem. In the simplest case, $\sigma_{j^{\prime }}^{2}$ coincides with the definition of global variance $\sigma_{j}^{2}$ in HARP [13], where the values consist of all the *j*^*th *^dimensional values across the dataset. With our genome vectorization strategy, the dataset contains two types of values: relative positions and absolute positions (refer to genome vectorization), which suggests that there are two groups of associated values across all the dimensions. The merge score (MS) between two clusters $C_{i_{1}}$ and $C_{i_{2}}$ is then defined as follows:

(1) $$MS\left(C_{i_{1}},C_{i_{2}}\right)=\sum_{j\in J_{i_{1}i_{2}}}R_{i_{1}i_{2}j}^{*},\text{ for all }R_{i_{1}i_{2}j}^{*}>t$$

(2) $$R_{i_{1}i_{2}j}^{*}=1-\frac{\left[2\left({\hat{\sigma }}_{i_{1}j}^{2}+{\hat{\sigma }}_{i_{2}j}^{2}\right)/m_{sj}+2\delta^{2}\right]}{\sigma_{j^{\prime }}^{2}}$$

(3) $$\delta =\left({\hat{\mu }}_{i_{1}j}-{\hat{\mu }}_{kj}\right)/\left(m_{i_{1}j}-m_{kj}\right)-\left({\hat{\mu }}_{i_{2}j}-{\hat{\mu }}_{kj}\right)/\left(m_{i_{2}j}-m_{kj}\right)$$

(4) $${\hat{\sigma }}_{ij}^{2}=m_{ij}\sigma_{ij}^{2}\text{ and }{\hat{\mu }}_{ij}=m_{ij}\mu_{ij}$$

where $J_{i_{1}i_{2}}$ is the intersecting set of dimensions between $C_{i_{1}}$ and $C_{i_{2}}$*t *is the cluster tightness threshold defined by the user, *m*_*ij *_is the number of data in set *i *at dimension *j*, subscripts *s *and *k *refer to the union and intersecting set of data between $C_{i_{1}}$ and $C_{i_{2}}$ respectively. R* is larger (at most 1) when the data in $C_{i_{1}}$ and $C_{i_{2}}$ are closer. A dimension *j *will be included by the subspace of the merged cluster if and only if $R_{i_{1}i_{2}j}^{*}>t$. The threshold *t *takes value between *0 *(loose clusters) and *1 *(tight clusters). The potential bias due to overlapping data is handled by the *δ *term. The weighted variance ${\hat{\sigma }}^{2}$ is to handle imbalanced cluster size. Other aspects of the merge score are described in Yip, et al. (2004) [13].

#### B) Algorithmic procedure for overlapping clustering

A simple agglomerative hierarchical approach to overlapping subspace clustering is to always retain the merging clusters. With such an approach, however, the number of clusters would grow exponentially with the number of data – complexity *O*(2^*M*^) where *M *is the number of data. Additionally, clusters overlapping with each other may be dependent on each other, in which case merging between them is not necessary and the computation of their merge scores adds unnecessary *computational burden*. Moreover, if a cluster has a subspace *S*, then the same set of data can always form a cluster in a space *S*' ⊂ *S*, which leads to *clustering ambiguity*. Finally, a subspace cluster can be obtained by merging its constituting clusters in many different ways. If the merging mechanism is not controlled, then the algorithm can generate a lot of *redundant clusters*.

**Figure 3:**
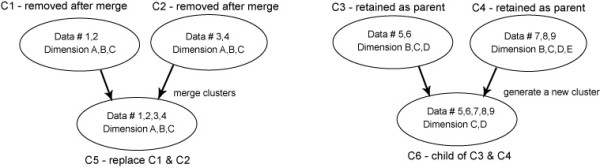
**Illustration of cluster merging and new cluster generation.** Clusters C1 and C2 are merged to form cluster C5 because Data 1–4 show similarity in all Dimensions A, B and C (no dimensional reduction). C3 and C4 combine to generate C6 as a new child cluster because Data 5–9 show similarity only in Dimension C and D.

Any hierarchical overlapping clustering algorithms should address the above issues. O-HARP's main contribution lies in its overlapping capability. The following notions are defined: If two clusters are combined to form a new cluster and are discarded afterwards, they are said to have *merged*. If they form a new cluster and are retained afterwards, they are said to have *generated *a new *child *cluster and are referred to as the *parents *of the new child cluster. Two clusters are merged if there is no dimensional reduction in the resulting cluster; otherwise a new cluster is generated (see Figure 3). These definitions imply the following two conditions: i) the set of data in a child cluster is a superset of the data in its parent clusters and ii) the set of dimensions in a child cluster is a subset of the data in its parent clusters. The algorithmic procedure is listed in Table 2 (a more detailed pseudo code can be found in Additional File 1). Line L1 is referred to as the *d loosening *mechanism, a concept borrowed from HARP [13]. The purpose is to start matching clusters with large subspaces first, and allow smaller subspaces and forbid larger subspaces in later iterations. This mechanism effectively maximizes the subspace between two merging clusters and prevents them from forming multiple ambiguous clusters in different subspaces. Moreover, this outermost loop indicates that computational complexity is linearly proportional to the number of dimensions, suggesting that the algorithm is favorable for high dimensional data. BuildScoreCache at line L2 refers to computing and storing in memory all MS scores larger than threshold *t *and that have a subspace with *d *dimensions. As mentioned previously, simply pairing up all clusters and computing their MS scores is not practical. Whether a potential match (i.e. merge or new cluster generation) is allowed is controlled within BuildScoreCache by the following rules:

**Table 2:** The algorithm of O-HARP. L1-L10 are line numbers.

Algorithm O-HARP (N is the number of dimensions in the dataset, t and f are the merge score and filtering thresholds)	
L1	For *d *: = *N *to 1 do {
L2	BuildScoreCache(*d*, t)
L3	While cache is not empty {
L4	FindBestMatch()
L5	PerformMatch()
L6	UpdateParentChildRelationship()
L7	UpdateScoreCache()
L8	}
L9	}
L10	FilterOutInsignificantClusters(f)

R1. Each pair of clusters can only have 1 child (yet each cluster can still have multiple children).

R2. Clusters in a family line cannot merge or generate new cluster with each other.

R3. All the clusters in a subspace must be disjoint (they can only overlap in different subspaces).

Rule R1 is used to prevent the same subset of data from forming separate clusters in different subspaces and hence to avoid the formation of ambiguous clusters. Moreover, having multiple child clusters from the same set of parents can potentially lead to redundant clusters. As child clusters inherit data and subspace from their parents, matching between clusters within the same family line is prohibited by R2 for the same reasons: to avoid ambiguous and redundant clusters. Rule R3 simply states that no fuzzy clustering [17] is allowed. This is because fuzzy clustering adds computational complexity and is unnecessary for the genome clustering problem.

After the function BuildScoreCache is performed, the memory (cache) holds information for all matching pairs that satisfy *t*, *d *and *R1*-*R3*. The best candidate pair is selected and removed from the cache repeatedly until all possible matches are performed (see L3 in Table 2). The best candidate pair is defined as the youngest descendant clusters of the pair with the highest MS score that shares the same subspace (implemented in L4). This is such that cluster coverage is maximized with the given threshold *t*; matching the highest MS-score pair forgoes future opportunities of matching with its child clusters, leading to suboptimal cluster coverage. After each match, the ancestor-descendant (parent-child) relationships among the clusters are updated, and all MS entries that are associated with the new update must be revised and checked for rule compliant again (L5-L7). After the core loop, L10 in Table 2 is used to filter out clusters that are insignificant with respect to i) the number of data and dimensions in the clusters and ii) similarity against their parent or child clusters, as detailed below.

i) Given a filtering threshold 0 ≤ *f *≤ 1, a cluster is considered to have a significant number of data and dimensions if and only if the condition (5) defined below holds true

(5) *αm *+ (1-*α*)*n *≥ *f*

(6) *α *= *N*/(*M *+ *N*)

where *m *and *n *are the number of data and the number of dimensions in the cluster respectively, and *M *and *N *the total number of data and dimensions in the dataset. The role of *α *is to handle any bias caused by the discrepancy between *M *and *N*. At *f *= 0, all clusters are considered significant.

ii) Given two clusters *C *and *C*' with an ancestor-descendant relationship, their similarity index (SI) is defined as:

(7) *SI*(*C*, *C*') = *αm *+ (1 - *α*)*n*

(8) *m *= |*d*_*C *_- *d*_*C*_|/max(*d*_*C*_, *d*_*C'*_)

(9) *n *= |*i*_*C *_- *i*_*C'*_|/max(*i*_*C*_, *i*_*C'*_)

where *d *is the number of data and *i *the number of dimensions of the subscripted cluster, *α *has the same definition as in equation (6). Using the same threshold *f *as before, the two clusters are regarded as significantly different if and only if *SI *≥ *f*. Based on this rule, we are able to extract only a subset of clusters that are significantly different from each other in terms of their data and dimensions.

Time complexity of O-HARP is *O*(2*M *× *c*(*HARP*)), where *c*(*HARP*) = *M*^2^(*N*^2 ^+ log^2^*M*) is the complexity of the non-overlapping version of HARP developed by Yip *et al*. (2004) [13]. The multiplication factor 2*M *is the number of clusters that O-HARP converges to given *t *= 0 (worst case). This increased complexity is however not a practical concern for the genome clustering problem, as *M *would be limited by the number of genomes deposited in the database. In the application on 441 phage genomes (see later section), where *M *= 441 and *N *= 8,001 (made up from 126 gene groups), the running time on a Pentium IV, 2.8 GHz single CPU machine is ~20 minutes.

### Algorithm Evaluation

#### A) Existing algorithms considered

In order to evaluate O-HARP, we compared its performance on the genome clustering evaluation data set against a selection of other algorithms. The selection is based on a number of properties: i) reported performance, ii) popularity determined by the number of citations, iii) recency, iv) availability of implementation, as well as v) problem relevance. All the selected algorithms are clustering-based algorithms for comparability; a tree-based method is compared and reported separately in the next section.

Table 3 shows the selected algorithms and the parameters with which they have been tested on our evaluation dataset. HARP represents a group of subspace clustering algorithms including PROCLUS, ORCLUS and FastDOC, as it embodies the essential characteristics of these algorithms such as disjoint and Euclidean-distance-based clustering. HARP was included for algorithmic evaluation because it has the best performance among this group of algorithms and is the developmental basis of our proposed algorithm. Cheng-Church [18] and SAMBA [19] are two popular algorithms that were originally designed for clustering analysis of microarray data, at which good performances have been observed. Although targeted at microarray data, Cheng-Church functions with a general principle that strives for consistent values among rows and columns of a subspace cluster. This general principle suggests that Cheng-Church may produce reasonable results on our genome clustering problem. In contrast, SAMBA works on a more limiting principle, where subspace clusters are formed based solely on the signs of data. Nevertheless, we believe it is of general bioinformatics interest to assess the performance of these popular biclustering algorithms on the genome clustering problem. SynFPS [5] is not a subspace clustering method but was designed to cluster genomes based on gene distribution, and is therefore included for comparison. The n-gram clustering method [6] was developed to classify species based on frequencies of short nucleotide sequences. This is the only method included in our comparison that uses no gene distribution information; it was included for evaluation because its target application, genome clustering, is highly similar to ours. HARP, SynFPS and n-gram produce only disjoint clusters. Therefore, their evaluation will be based on the seven disjoint clusters of our evaluation dataset only.

**Table 3:** Evaluated algorithms and the range of parameters that have been tested.

		Test range			
			
Algorithm	Parameter	Min.	Max.	Step size	Best case
O-HARP	*t *– cluster tightness	0.1	0.9	0.04	**0.58**
	*f *– filtering threshold	0	0.04	0.8	**0.24**
HARP	*K *– target # of clusters	3	30	1	**7**
	*MOP *– max. outlier percentage	5	13	1	**9**
SAMBA	*v *– version	(discrete: 6 versions – tested all)			**v2**
	*t *– try covering all probes	(discrete: true/false – tested both)			**true**
	*f *– overlap factor	0.001	0.13	varies	**0.03**
	*rp *– responding probes to hash	3	30	3	**9**
Cheng-Church	*d *– delta	0.03	0.9	varies	**0.07**
	*a *– alpha	1.0	1.2	0.1	**1.2**
	*K *– target # of clusters	10	300	varies	**70**
FastDOC	*K *– target # of clusters	3	18	3	**n/a**
	*b *– beta	0.2	0.45	0.05	**n/a**
	*w *– cluster width	0.05	0.65	0.2	**n/a**
	*MAXITER *– max. # of inner iterations	8000	10000	2000	**n/a**
SynFPS	*K *– target # of clusters	5	10	1	**9**
n-gram	*n *– length of nucleotide sequence	2	6	1	**5**
	*k *– target # of clusters	3	10	1	**9**

#### B) Results

The performances of O-HARP and other algorithms are shown in Table 4. O-HARP has the best SCE, CI and DNC scores, which indicate that it has the best ability to detect consensus gene distribution patterns (implied by its detected subspaces) and genome clusters without including excessive unnecessary clusters. O-HARP's RCE score is however lower than those of SynFPS and HARP. This means that without considering the subspace correctness, the data are grouped better in SynFPS and HARP. By looking at individual RCE scores for each cluster, we find that the average RCE across the disjoint clusters for O-HARP, HARP and SynFPS are 0.25, 0.35 and 0.10 respectively, indicating that O-HARP actually has a better RCE than HARP when only the disjoint clusters are considered. Consequently, we may only conclude that applying SynFPS and HARP on a disjoint problem produces better results than applying O-HARP on an overlapping problem. The major drawback of SynFPS and HARP is their inability to produce overlapping clusters. On the other hand, n-gram produces a poor RCE score even when only the seven disjoint clusters are considered. Such incompatibility between n-gram's clustering and the model clustering (i.e. evaluation data) suggests that frequent short nucleotide sequences encode no information about gene distribution.

**Table 4:** Performances of different algorithms on the evaluation data set.

Algorithm	Subspace clustering error (SCE)	Row clustering error (RCE)	Coverage index (CI)	Discrepancy in the number of clusters (DNC)
O-HARP	**0.38**	0.37	**0.38**	1
HARP	0.49	0.31	0.47	3
SAMBA	0.96	0.81	0.96	10
Cheng-Church	0.78	0.60	0.77	19
SynFPS	n/a	**0.13**	n/a	2
n-gram	n/a	0.37	n/a	2

HARP, SAMBA and Cheng-Church produce low SCE scores for different reasons. HARP produced higher RCE scores than SCE scores for 6 out of the 7 disjoint clusters when the clusters are individually analyzed, indicating that its performance bottleneck is in subspace identification rather than data grouping. This weakness is mainly caused by its definition of the dimensional global variance, the improved version of which is employed by O-HARP. SAMBA uses a probabilistic model to detect up/down regulation in gene expression data [19]. As expected, the model does not generalize to our problem and failed to detect 10 out of 13 clusters from our evaluation dataset, which is the main reason for a low SCE. Cheng-Church uses a similarity score called the mean squared residue to detect coherent rows and/or columns in a dataset [18]. This model is able to capture Euclidean-based similarity, as required by our problem. Consequently, the performance is better than SAMBA. However, it tends to include excessive dimensions and data in the clusters, causing a relatively low SCE score. It is noteworthy to restate that we included Cheng-Church and SAMBA for performance comparison because of their popularity for biclustering in bioinformatics research.

O-HARP detected four out of 13 clusters poorly. However, as we lowered the filtering threshold *f*, we found that these clusters could in fact be correctly identified: at *f *< 0.15, the CI scores produced by O-HARP are close to perfect (~0.07) while *SCE *≈0.6. This suggests that O-HARP can identify all the clusters in the evaluation data set, but there is not a single threshold value that can produce the ideal filtering across all clusters. Nevertheless, it is arguable whether such a single threshold is necessary or feasible because the model clustering of an unsupervised learning problem, upon which algorithms are evaluated, is inevitably subjective.

### Similarities and new perspectives against the Phage Proteomic Tree

**Figure 4:**
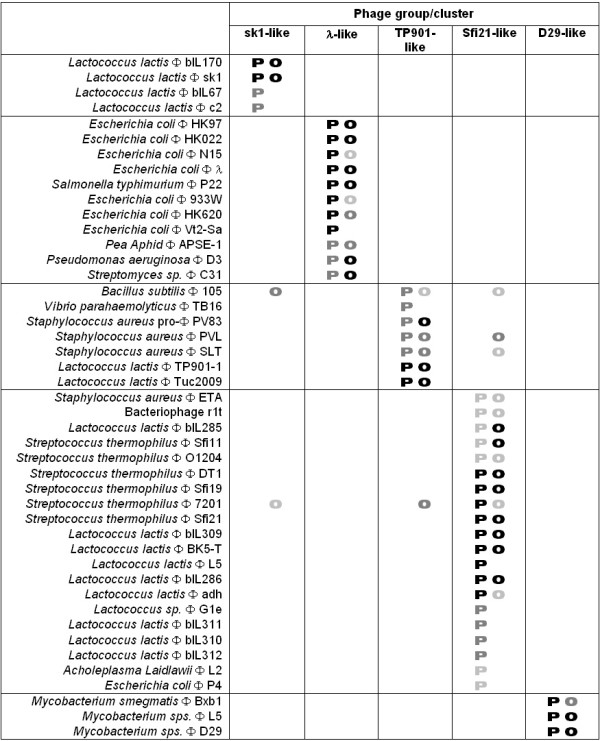
**Comparison of phage clusters between PPT ("P") and O-HARP ("O").** A darker color indicates a stronger association between the phage and the phage group. Association strength is determined by proteomic distance for PPT and gene-distribution distance for O-HARP. For instance, phage bIL170 has a darker grey than bIL67 in the sk1-like group for the alphabet letter "P" because it has a closer proteomic distance to phage sk1. Phage 933W has a light grey for "O" because its gene distribution (computed by O-HARP) is not as close to phage *λ *as some other phages such as HK97 and P22. Abbreviation: Φ – Bacteriophage.

We further compared O-HARP to the Phage Proteomic Tree (PPT) [12] to validate its biological significance. The PPT utilizes sequence distances among the predicted proteome of phages to function as a genome-based taxonomical system. With PPT, Rohwer and Edwards showed the relationship of 105 phages with an unrooted tree and classified the genomes into related phage groups based on their proteomic distances [12]. Phage groups of *Siphoviridae *(sk1-like, *λ*-ike, TP901-like, sfi21-like and D29-like) consist of a total of 45 phages, representing a significant portion of the total number of phages analyzed [12]. The Siphophage groups deduced from PPT, along with the clustering results generated by O-HARP over the same 105 phages, are illustrated in Figure 4. The strength of association between a phage and a phage group is shown by different levels of grey, and is determined by the proteomic distance in the case of the PPT and by the difference in gene distribution in the case of O-HARP.

**Figure 5:**
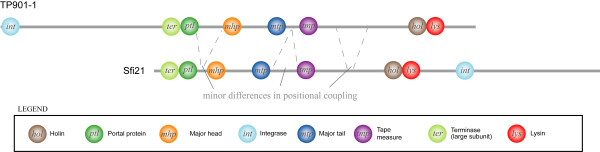
Similarity in genomic structure between bacteriophage TP901-1 and Sfi21.

Figure 4 shows an overall agreement between the two approaches to phage classification. The more remarkable differences come from *Bacillus subtilis *phage 105 and *Streptococcus thermophilus *phage 7201, which are classified as TP901-like and Sfi21-like respectively by PPT, but are equally clustered across three phage groups by O-HARP. O-HARP weakly associated phage 105 as TP901-like because of the absence of the integrase gene, which is highly positionally conserved among the other members of the group. Consequently, although there is strong resemblance in other genes in terms of genomic distribution, phage 105 was found in a child cluster to the core TP901-like cluster, instead of the TP901-like cluster itself. The same explanation also applies to the weak classification of phage 7201 as Sfi21-like. Phage 105 is also classified as Sfi21-like and sk1-like because it showed relatively strong resemblance in the distribution of a set of genes including the terminase, portal, tape measure, holin and lysin. Likewise, phage 7201 is classified as sk1-like and TP901-like because of a distribution resemblance over the similar gene set. These observations suggest that the genomic structures of Sfi21-like, TP901-like and sk1-like phages do share a certain degree of similarity over a non-trivial set of genes. The genomic position of the structural genes of Sfi21 and TP901-1 are illustrated in Figure 5; phage sk1 also has similar gene arrangements. The comparison shows that the genomes are highly similar when they are circularized (more discussion on circular genomes is provided in the next section). In fact, Sfi21-like, TP901-like and sk1-like phages coexist in the same descendant subspace cluster when the requirement of absolute genomic position similarity is relaxed. This suggests that, unlike the *λ*- and D29-like phage groups, the Sfi21-, TP901- and sk1-like phage groups might be validly considered as subgroups of a more generic group.

O-HARP predicted phage groups that are compatible to the PPT (and hence the ICTV classification system [11]). Additionally, it enabled the genomes to be analyzed from the perspective of gene distribution, augmenting existing knowledge of phage relationships. Another advantage of O-HARP over the PPT is that it can cluster uncompleted (partial) genomes by matching local genomic arrangements, in contrast to the method of proteomic distance which requires genomes to have comparable sizes for an unbiased measurement.

### Four common gene arrangements detected in 441 phage genomes

**Figure 6:**
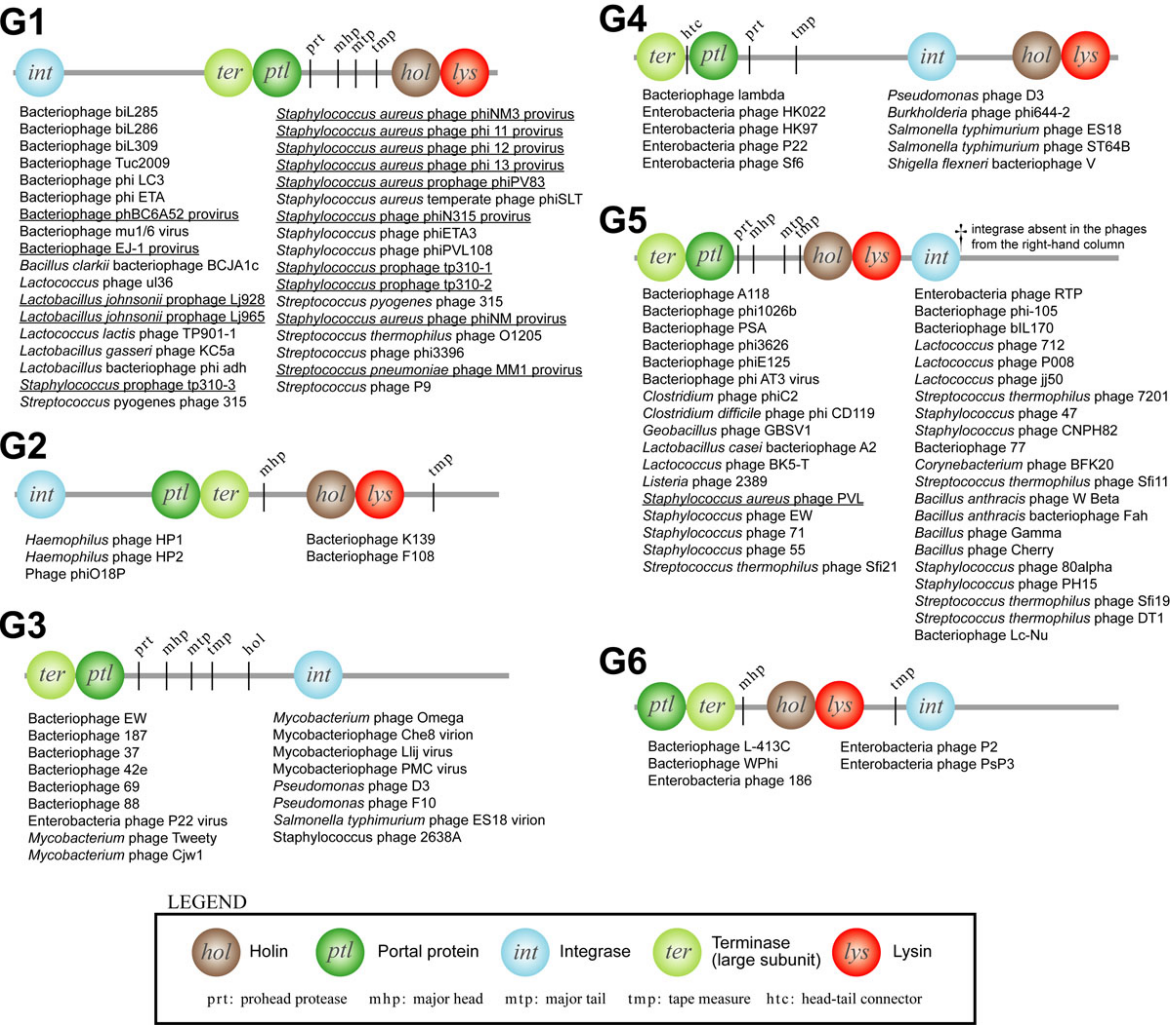
**Common gene order patterns for tailed phages, labeled with G1-G6.** Listed under each pattern are examples of phage members for that pattern. Prophages are underlined. The strongly positionally conserved genes are depicted with circles. The inclined gene symbols (e.g. prt) depict genes that are conserved in terms of position and existence among most, but not all, of the phage members.

We applied O-HARP to 441 phage genomes, comprising all the complete phage genomes and 23 prophage genomes available from NCBI as at December 2007 [20]. Six clusters that are associated with the rearrangement of integrase, terminase, holin, lysin and portal protein are illustrated in Figure 6. These five genes are selected because they are found to be strongly conserved in position, as determined by the number of members in their associated subspace clusters generated by O-HARP. The other genes that are found conserved in the neighboring ancestor and descendant clusters, including structural genes major head, major tail and tape measure, are also illustrated in Figure 6.

The four more notable gene distribution patterns are *G1*, *G3*, *G4 *and *G5*, comprising a total of 100 bacteriophages. This indicates that the gene order for integrase (*int*), terminase (*ter*) and portal (*ptl*) is highly conserved in two distinct arrangements among the observed phage population: *int*-*ter*-*ptl *for *G1 *and *ter*-*ptl*-*int *for *G3*, *G4 *and *G5*. Holin and lysin are arranged in various positions, which may be associated with different functional strategies for leaving hosts in terms of timing control [21].

Gene order pattern *G3*, which lacks holin and lysin genes, consists of phages that infect distantly related hosts, including phyla *Actinobacteria *(*Mycobacterium*), *Proteobacteria *(*Pseudomonas*) and *Firmicutes *(*Staphylococcus*). Because of the absence of holin and lysin, members in the *G3 *are rather more diverse than the two similar gene order patterns *G4 *and *G5*, in which both have more specific hosts, *Proteobacteria *and *Firmicutes*. Between these two groups, the location of the holin/lysin genes becomes a striking feature, in which the holin/lysin genes are positioned outside of the *ter*-*ptl*-*int *group in *G4*, whereas in *G5 *they are placed between the *int *gene and the *ter*-*ptl *group.

*G6 *consists of only P2-like bacteriophages (*Myoviridae*), having a terminase gene at one end of the genome and integrase in the middle. A comparison between *G6 *and *G2 *reveals that the gene arrangement of *int*-*ptl*-*ter-hol *appears to be highly similar if the genomes are circularized. In fact, pattern *G2 *contains *Haemophilus *HP1 and HP2, which have been shown to possess similar genes to bacteriophage P2 and have been literally and taxonomically grouped into the P2-like genus [22]. It is however noteworthy that these P2-like phages all contain unique genes, some with unknown functions. P2-like phages, including members of *G2 *and *G6*, normally appear in a linear form of double-stranded DNA in their life cycles, but appear in a circular form during DNA replication in the lytic cycle [23]. Patterns *G2 *and *G6 *being similar in gene arrangement and appearing in circular form during replication indicate that their differentiation is caused by different cleavages to the circular DNA during replication. The difference in cleavage sites may in turn be a result of natural selection or spontaneous mutation. An analogous consideration can be applied to the comparison between *G1 *and *G5*, which is similar to the contrast between TP901-like and Sfi21-like phages discussed previously. However, regardless of the difference in cleavage sites, *G2 *and *G6 *(*G1 *and *G5*) actually formed a single cluster at a descendant node where the dimensions associated with absolute positioning of genes are excluded. This feature of O-HARP helps avoid biases that arise from arbitrary start points of circular genomes (14% of the phages shown in Figure 6 have circular genomes – see phage details in Additional File 1).

The current phage taxonomical system has been discussed recently and new genera for *Siphoviridae *have been proposed [24,25]. One of the proposed genera was 'Sfi21-like' and eight completely sequenced phages were originally proposed by Brüssow and Desiere as members of that genus [24]. Six out of these eight phages agree with our observation in pattern *G5 *(see Table 5). The two others (phage adh and 7201), although having close proteomic distance to Sfi21-like phages, have their terminase-portal gene cluster located in the middle while holin and lysin genes are located towards the end of the genome, contradicting with *G5*. These two phages, in contrast to the other members, would have evolved with a holin-lysin gene translocation event. This indicates that there is a subgroup of Sfi21-like phages comprising a relatively divergent genome organization, and that 'Sfi21-like' should be a multi-group population rather than a group (genus).

**Table 5:** Phage members of the Sfi21-like genus.

	Brüssow and Desiere	O-HARP	PPT
*Streptococcus thermophilus *Φ Sfi21	∙	∙	∙
*Streptococcus thermophilus *Φ Sfi19	∙	∙	∙
*Streptococcus thermophilus *Φ DT1	∙	∙	∙
*Lactococcus lactis *Φ BK5-T	∙	∙	∙
*Staphylococcus aureus *Φ PVL	∙	∙	
*Bacillus subtilis *Φ 105	∙	∙	
*Lactococcus lactis *Φ adh	∙	∘	∙
*Streptococcus thermophilus *Φ 7201	∙	∘	∙
*Staphylococcus aureus *Φ SLT		∘	
*Staphylococcus aureus *Φ ETA		∘	∘
Bacteriophage r1t		∘	∘
*Lactococcus lactis *Φ bIL285		∙	∘
*Streptococcus thermophilus *Φ Sfi11		∙	∘
*Streptococcus thermophilus *Φ O1204		∘	∘
*Lactococcus lactis *Φ bIL309		∙	∙
*Lactococcus lactis *Φ bIL286		∙	∙
*Staphylococcus *Φ 71		∙	
*Listeria *Φ A118		∙	
*Listeria *Φ 2389		∙	
*Lactobacillus *Φ phi AT3		∙	
*Lactobacillus *Φ A2		∙	
*Clostridium *phi3626		∙	
*Listeria monocytogenes *Φ PSA		∙	
*Geobacillus *Φ GBSV1		∙	
*Staphylococcus *Φ phiPVL108		∙	
*Lactococcus sp*. Φ G1e			∙
*Lactococcus lactis *Φ bIL311			∙
*Lactococcus lactis *Φ bIL310			∙
*Lactococcus lactis *Φ bIL312			∙
*Acholeplasma Laidlawii *Φ L2			∙
*Escherichia coli *Φ P4			∙

Comparison among the results proposed by Brüssow and Desiere [24], Phage Proteomic Tree [12], and O-HARP. A solid circle corresponds to strong evidence for a phage being a member of Sfi21-like; an outlined circle corresponds to weaker evidence. Abbreviation: Φ – Bacteriophage.

Our observation also suggests that there are nine new members of the group 'Sfi21-like' according to their organizational similarity in the selected genes. These nine members are *Staphylococcus *71, phiPVL108, *Listeria *A118, 2389, *Lactobacillus phi *AT3, A2, *Clostridium *phi3626, *Geobacillus *GBSV1, and *Listeria monocytogenes *PSA (see Table 5). Besides sharing a similar landmark-like gene organization, these phages also infect closely related hosts in terms of phylogenetic relations. These phages, however, were not detected by the PPT as Sfi21-like. Our results herein imply that gene distribution information might provide a new perspective on the phage classification system.

## Discussion

### Relevance and incompatibility of other computational methods

Inspecting species relationships based on gene distribution utilizes information about gene co-occurrence, gene order, gene-to-gene distances and absolute gene positions in the genome. Many works have contributed to evolutionary knowledge by manually inspecting species from the same lineage based on gene distribution [24,26-29]. Nevertheless, the use of computational methods is necessary to tackle the rapidly increasing amount of genome data. Although a number of computational methods related to gene distribution and genome rearrangement exist, most of them are not capable of clustering whole genomes based on genomic-context information, hence the development of this work. More specifically, existing methods such as ADHoRe [30], EM_TRAILS [31] and EDE [32] have been designed for analyzing closely related species and are only capable of handling genes that are common in all the genomes being compared; genes not shared by any one of the genomes must be removed prior to analysis [33]. Many other computational comparative genomics methods related to gene rearrangement are also limited by the requirement that the species being compared must be closely related [5,34]. While these methods are evidently valuable for the analysis of mammalian genomes, they are not capable of a large-scale, high-level analysis of microbial genomes where wide samples across distant species are analyzed collectively. Large-scale, high-level analyses are however indispensable for revealing evidence concerning evolution and diversity within a population [35]. Other methods related to gene distribution are Grappa [36], MGR [33] and the genomic-structure conservation approach [37]. These methods, as well as EDE, are aimed at phylogenetic tree reconstruction, which completeness in describing evolutionary relationships among microbial species is still debated [2-4]. Methods such as Larmarck [38], the P-quasi complete linkage approach [39] and ADHoRe provide clustering techniques to predict operons and collinear genomic blocks among multiple species, but they provide no clear linkage to phylogenetic inference and cannot classify or cluster genomes. In human genomics research, there is one group of methods that aim to align conserved regions and produce mapping among multiple genomes. Examples are SLAGAN [40], Mauve [41] and MAGIC [34]. These methods are related to the genome clustering problem discussed in this paper, in the sense that they also identify genomic segments that exhibit similar gene distribution patterns. However, their approaches are targeted at the visual inspection of genome rearrangement among a few related species and provide no deterministic strategies for clustering a large number of genomes based on the conserved patterns.

The methods described above are relevant but are incompatible with our problem because they provide no means for genome clustering. Consequently, the methods that we were able to include for algorithm evaluation were limited. In this work, we are pursuing a method that solves a genomics problem currently under-addressed in computational biology. This problem, namely the clustering of genomes based on genomic context, is becoming more important as the number of sequenced genomes increases rapidly.

### Significance of gene-distribution based clustering for phages

Phages are taxonomically classified based on the physical characteristics of their virions, genome size and type; however, no taxonomic levels below that of family can be defined with this classification system [42]. This complication can be explained by a number of recent observations on phage genomes: a large number of novel sequences, high genomic mosaicism, and genes being highly mobile, which have resulted in massive HGT [10,43]. However, while phage genomes are mosaic overall, subgroups of phages have often been observed with comparable genome structures. For example, gene order has been found to be strikingly conserved for structural and assembly genes in myco- and sipho- phages [43,44]. Their gene order remains strongly conserved even in the presence of high genetic mosaicism, where genes or gene clusters are shared among different phages in a reassorted or mosaic manner [10,45]. Such conservation may be caused by functional constraints, such as favoring lateral gene transfer [46] or allowing co-transcription and co-regulation of genes [43,46]. Conservation of gene positions and relative positions between genes has also been observed [47,48]. It has been suggested that such positional conservation results from natural selection – although the recombination events that give rise to mosaicism can happen at random locations in the phage genomes, natural selection could eliminate unfit phages and let only those survive who have recombination joints at selected positions [10,45]. These observations suggest that gene distribution can provide valuable information for understanding phage relationships and allow alternative perspectives on phage classification, justifying the methods we propose in this paper.

### Future algorithmic improvement

O-HARP's ability to filter out subsets of dimensions enables additional biological features to be simply appended to the genome vectors without compromising the similarity measure on the original features. The biological features that deserve future investigations include transcriptional directionality of genes and the isoelectric point, hydrophobic region, and molecular weight of the gene products. These features can potentially enhance the underlying meaning of a cluster and provide further information for downstream analysis such as the prediction of function for novel gene groups.

## Conclusion

We have proposed the use of an overlapping subspace clustering algorithm to assist evolutionary study through objectively classifying genomes based on their resemblance in gene order, gene content and genome positions. The advantage of subspace clustering over traditional clustering is the ability to associate clusters with gene arrangement patterns, preserving genomic information in the clusters produced. Additionally, overlapping capability is desirable for the discovery of multiple conserved patterns within a single genome, such as those acquired from different species via HGT. We presented O-HARP and demonstrated its significance through evaluation and application to bacteriophage genomes. The phage clusters were compatible overall with the Phage Proteomic Tree and the ICTV classification system, and have enabled additional observations on Siphophage genomics through an alternative perspective derived from gene distribution conservation. In general, the proposed method is suitable for application to genomes with high genetic exchange and various conserved gene arrangement, and is potentially exploitable for the detection of prophages in bacterial genomes.

## Methods

### Evaluation data

For evaluation, we manually composed a data set that captures several complexities that reflect real data. Firstly, regarding overlapping complexity, the genomes are chosen such that some of them belong to a single group and some to multiple groups. The data set contains seven disjoint clusters, upon which six overlapping clusters are built hierarchically, totaling 13 clusters. Secondly, to capture the complexity in gene distribution, we included various genomes that contain the same set of genes coexisting in different distributions. We also included distributions that have the same gene order with varying gene-to-gene distances. Thirdly, in order to test the algorithm's ability to distinguish between absolute and relative gene distributions, we included cases where the distributions are based only on gene-to-gene distances and cases where the distributions are based on both distances and positions. Finally, we included outliers to serve as noise. The genes that we selected to include in the evaluation data set are major head, major tail, tape measure, prohead protease, integrase, terminase, portal, holin and lysin genes. They were selected because of their prevalent existence; many of these genes appear to be common in *Siphoviridae *[24], which constitutes the largest proportion of the observed DNA phage population. The data is available for download from the project website [9].

### Performance measures

The performance of a clustering algorithm is evaluated by comparing its generated clusters with the model clusters. We employ a performance measure referred to here as Subspace Clustering Error (SCE) [8], which formula is summarized as follows: Let the model clustering be *S *and the clustering generated by a target algorithm be *S*'. The clusters in *S *are matched against the clusters in *S' *and the number of identical *elements *between each pair of matching clustering are totaled. The sum is denoted as *D*_max_. The union set of *elements *between *S *and *S' *is denoted by *U*, and the number of elements |*U*|. For computing SCE, an "element" in a cluster corresponds to one datum and one dimension (which can be thought of an element in a matrix). We use subscript "*S*" to denote this:

(10) $$SCE(S,S^{\prime })=\frac{\left|U_{S}\right|-D_{S_{\max}}}{\left|U_{S}\right|}$$

For traditional clustering, a measure only needs to tell how well data are grouped, as there is no subspace information. We also employ such a measure in our evaluation, called Row Clustering Error (RCE), in order to compare non-overlapping algorithms as well as to provide auxiliary information about algorithmic performances for the overlapping ones. For RCE, an "element" in *U *and *D*_max _corresponds only to one datum. We use subscript "*R*" to denote this.

(11) $$RCE(S,S^{\prime })=\frac{\left|U_{R}\right|-D_{R_{\max}}}{\left|U_{R}\right|}$$

In subspace clustering, algorithms tend to generate more clusters than necessary because of a large number of possible intrinsic subspaces. Therefore, we introduce two more measures, the Coverage Index (CI) and the discrepancy in the number of clusters (DNC). If the number of clusters in *S*' is larger than that in *S*, then after the matching of *S *and *S'*, a subset of clusters from *S' *will become redundant. We denote this subset *J*. Then CI is defined as *CI*(*S*, *S*') = *SCE*(*S*, *S*'\*J*), where *S*'\*J *indicates set *S' *excluding set *J*. DNC is simply the difference in the number of clusters between *S *and *S'*, or the number of redundant clusters. For SCE, RCE and CI, a score of 0 means perfect and 1 means worst. For DNC, a value close to zero is also preferred.

## List of abbreviations

dsDNA, double-stranded deoxyribonucleic acid; SCE, Subspace Clustering Error; RCE, Row Clustering Error; CI, Coverage Index; DNC, Discrepancy in the Number of Clusters; PPT, Phage Proteomic Tree; ICTV, International Committee on Taxonomy of Viruses.

## Authors' contributions

JL conceived of the research and drafted the manuscript. SKH participated in validating the integrity of the algorithm as well as results evaluation. S–LT provided expertise in bacteriophage analysis and data interpretation. All authors have participated in preparing the manuscript, have read and approved the final manuscript.

## Supplementary Material

Additional File 1 Supplementary Information. Pseudo-code for HOSC and details for the phages listed in Figure 6. Click here for file

## References

[B1] Delsuc F Brinkmann H Philippe H Phylogenomics and the reconstruction of the tree of life Nat Rev Genet 2005 6 5 361 375 15861208 10.1038/nrg1603

[B2] Wolf YI Rogozin IB Grishin NV Koonin EV Genome trees and the tree of life Trends Genet 2002 18 9 472 479 12175808 10.1016/s0168-9525(02)02744-0

[B3] Gogarten JP Townsend JP Horizontal gene transfer, genome innovation and evolution Nat Rev Microbiol 2005 3 9 679 687 16138096 10.1038/nrmicro1204

[B4] Zhaxybayeva O Lapierre P Gogarten JP Genome mosaicism and organismal lineages Trends Genet 2004 20 5 254 260 15109780 10.1016/j.tig.2004.03.009

[B5] Li J Halgamuge S Kells C Tang S-L Gene function prediction based on genomic context clustering and discriminative learning: an application to bacteriophages BMC Bioinformatics 2007 8 Suppl 4 S6 17570149 10.1186/1471-2105-8-S4-S6 PMC1892085

[B6] Tomovic A Janicic P Keselj V n-gram-based classification and unsupervised hierarchical clustering of genome sequences Comput Methods Programs Biomed 2006 81 2 137 153 16423423 10.1016/j.cmpb.2005.11.007

[B7] Parsons L Haque E Liu H Subspace clustering for high dimensional data: a review SIGKDD Explor Newsl 2004 6 1 90 105

[B8] Patrikainen A Meila M Comparing Subspace Clusterings IEEE Transactions on Knowledge and Data Engineering 2006 18 7 902 916

[B9] The O-HARP Project Website http://www.mame.mu.oz.au/bioinformatics/hosc

[B10] Hendrix RW Bacteriophage genomics Curr Opin Microbiol 2003 6 5 506 511 14572544 10.1016/j.mib.2003.09.004

[B11] Nelson D Phage taxonomy: we agree to disagree J Bacteriol 2004 186 21 7029 7031 15489416 10.1128/JB.186.21.7029-7031.2004 PMC523225

[B12] Rohwer F Edwards R The Phage Proteomic Tree: a genome-based taxonomy for phage J Bacteriol 2002 184 16 4529 4535 12142423 10.1128/JB.184.16.4529-4535.2002 PMC135240

[B13] Yip KY Cheung DW Ng MK HARP: a practical projected clustering algorithm Knowledge and Data Engineering, IEEE Transactions on 2004 16 11 1387 1397

[B14] Aggarwal C Procopiuc C Wolf J Yu P Park. J A framework for finding projected clusters in high dimensional spaces ACM SIGMOD: 1999 1999

[B15] Aggarwal CC Yu PS Finding generalized projected clusters in high dimensional spaces ACM SIGMOD intl conf Management of data: 2000 2000 Dallas, Texas, United States: ACM Press 70 81

[B16] Procopiuc CM Jones M Agarwal PK Murali TM A Monte Carlo algorithm for fast projective clustering ACM SIGMOD intl conf Management of data: 2002 2002 Madison, Wisconsin: ACM Press 418 427

[B17] Doring C Borgelt C Kruse R Fuzzy clustering of quantitative and qualitative data 2004 81 84 89

[B18] Cheng Y Church GM Biclustering of Expression Data8th Intl Conf Intelligent Systems for Molecular Biology 2000 AAAI Press 93 103 10977070

[B19] Tanay A Sharan R Shamir R Discovering statistically significant biclusters in gene expression data Bioinformatics 2002 18 Suppl 1 S136 144 12169541 10.1093/bioinformatics/18.suppl_1.s136

[B20] GenBank Complete Phage Genomes National Center for Biotechnology Information 2007 http://www.ncbi.nlm.nih.gov/genomes/static/phg.html

[B21] Wang IN Smith DL Young R Holins: the protein clocks of bacteriophage infections Annu Rev Microbiol 2000 54 799 825 11018145 10.1146/annurev.micro.54.1.799

[B22] Williams BJ Golomb M Phillips T Brownlee J Olson MV Smith AL Bacteriophage HP2 of Haemophilus influenzae J Bacteriol 2002 184 24 6893 6905 12446640 10.1128/JB.184.24.6893-6905.2002 PMC135456

[B23] Nilsson AS Liungquist EH Calendar R The P2-like bacteriophages The bacteriophages 2006 2 Oxford Press 365 390

[B24] Brussow H Desiere F Comparative phage genomics and the evolution of Siphoviridae: insights from dairy phages Mol Microbiol 2001 39 2 213 222 11136444 10.1046/j.1365-2958.2001.02228.x

[B25] Proux C van Sinderen D Suarez J Garcia P Ladero V Fitzgerald GF Desiere F Brussow H The dilemma of phage taxonomy illustrated by comparative genomics of Sfi21-like Siphoviridae in lactic acid bacteria J Bacteriol 2002 184 21 6026 6036 12374837 10.1128/JB.184.21.6026-6036.2002 PMC135392

[B26] Blatny JM Godager L Lunde M Nes IF Complete genome sequence of the Lactococcus lactis temperate phage [phi]LC3: comparative analysis of [phi]LC3 and its relatives in lactococci and streptococci Virology 2004 318 1 231 244 14972551 10.1016/j.virol.2003.09.019

[B27] Tamames J Gonzalez-Moreno M Mingorance J Valencia A Vicente M Bringing gene order into bacterial shape Trends in Genetics 2001 17 3 124 126 11226588 10.1016/s0168-9525(00)02212-5

[B28] Kwan T Liu J DuBow M Gros P Pelletier J The complete genomes and proteomes of 27 Staphylococcus aureus bacteriophages Proc Natl Acad Sci USA 2005 102 14 5174 5179 15788529 10.1073/pnas.0501140102 PMC556006

[B29] Tuohimaa A Riipinen KA Brandt K Alatossava T The genome of the virulent phage Lc-Nu of probiotic Lactobacillus rhamnosus, and comparative genomics with Lactobacillus casei phages Arch Virol 2006 151 5 947 965 16328134 10.1007/s00705-005-0672-0

[B30] Vandepoele K Saeys Y Simillion C Raes J Van De Peer Y The automatic detection of homologous regions (ADHoRe) and its application to microcolinearity between Arabidopsis and rice Genome Res 2002 12 11 1792 1801 12421767 10.1101/gr.400202 PMC187543

[B31] Rogozin IB Makarova KS Murvai J Czabarka E Wolf YI Tatusov RL Szekely LA Koonin EV Connected gene neighborhoods in prokaryotic genomes Nucleic Acids Res 2002 30 10 2212 2223 12000841 10.1093/nar/30.10.2212 PMC115289

[B32] Wang LS Warnow T Moret BM Jansen RK Raubeson LA Distance-based genome rearrangement phylogeny J Mol Evol 2006 63 4 473 483 17021931 10.1007/s00239-005-0216-y

[B33] Bourque G Pevzner PA Genome-scale evolution: reconstructing gene orders in the ancestral species Genome Res 2002 12 1 26 36 11779828 PMC155248

[B34] Swidan F Rocha EP Shmoish M Pinter RY An integrative method for accurate comparative genome mapping PLoS Comput Biol 2006 2 8 e75 16933978 10.1371/journal.pcbi.0020075 PMC1526463

[B35] Field D Feil EJ Wilson GA Databases and software for the comparison of prokaryotic genomes Microbiology 2005 151 Pt 7 2125 2132 16000703 10.1099/mic.0.28006-0

[B36] Moret BM Wang LS Warnow T Wyman SK New approaches for reconstructing phylogenies from gene order data Bioinformatics 2001 17 Suppl 1 S165 173 11473006 10.1093/bioinformatics/17.suppl_1.s165

[B37] Blin G Chauve C Fertin G Genes Order and Phylogenetic Reconstruction: Application to -Proteobacteria3rd RECOMB Comparative Genomics Satellite Workshop: 2005; Dublin, Ireland 2005 11 20

[B38] Wolf YI Rogozin IB Kondrashov AS Koonin EV Genome alignment, evolution of prokaryotic genome organization, and prediction of gene function using genomic context Genome Res 2001 11 3 356 372 11230160 10.1101/gr.gr-1619r

[B39] Fujibuchi W Ogata H Matsuda H Kanehisa M Automatic detection of conserved gene clusters in multiple genomes by graph comparison and P-quasi grouping Nucleic Acids Res 2000 28 20 4029 4036 11024184 10.1093/nar/28.20.4029 PMC110780

[B40] Brudno M Malde S Poliakov A Do CB Couronne O Dubchak I Batzoglou S Glocal alignment: finding rearrangements during alignment Bioinformatics 2003 19 Suppl 1 i54 62 12855437 10.1093/bioinformatics/btg1005

[B41] Darling AC Mau B Blattner FR Perna NT Mauve: multiple alignment of conserved genomic sequence with rearrangements Genome Res 2004 14 7 1394 1403 15231754 10.1101/gr.2289704 PMC442156

[B42] Ackermann HW Calendar R Classification of Bacteriophages The Bacteriophages 2006 2 Oxford University Press 8 16

[B43] Hatfull GF Pedulla ML Jacobs-Sera D Cichon PM Foley A Ford ME Gonda RM Houtz JM Hryckowian AJ Kelchner VA Namburi S Pajcini KV Popovich MG Schleicher DT Simanek BZ Smith AL Zdanowicz GM Kumar V Peebles CL Jacobs WR Jr Lawrence JG Hendrix RW Exploring the mycobacteriophage metaproteome: phage genomics as an educational platform PLoS Genet 2006 2 6 e92 16789831 10.1371/journal.pgen.0020092 PMC1475703

[B44] Brussow H Hendrix RW Phage Genomics: Small Is Beautiful Cell 2002 108 1 13 16 11792317 10.1016/s0092-8674(01)00637-7

[B45] Pedulla ML Ford ME Houtz JM Karthikeyan T Wadsworth C Lewis JA Jacobs-Sera D Falbo J Gross J Pannunzio NR Brucker W Kumar V Kandasamy J Keenan L Bardarov S Kriakov J Lawrence JG Jacobs WR Jr Hendrix RW Hatfull GF Origins of highly mosaic mycobacteriophage genomes Cell 2003 113 2 171 182 12705866 10.1016/s0092-8674(03)00233-2

[B46] Tamames J Evolution of gene order conservation in prokaryotes Genome Biol 2001 2 6 RESEARCH0020 11423009 10.1186/gb-2001-2-6-research0020 PMC33396

[B47] Hendrix RW Bacteriophages: evolution of the majority Theor Popul Biol 2002 61 4 471 480 12167366 10.1006/tpbi.2002.1590

[B48] Recktenwald J Schmidt H The nucleotide sequence of Shiga toxin (Stx) 2e-encoding phage phiP27 is not related to other Stx phage genomes, but the modular genetic structure is conserved Infect Immun 2002 70 4 1896 1908 11895953 10.1128/IAI.70.4.1896-1908.2002 PMC127862

