# Supplementary material for: Genome classification by gene distribution: An overlapping subspace clustering approach
Source: BMC Evol Biol. 2008 Apr 23;8:116. doi: 10.1186/1471-2148-8-116 (PMC2383906; doi:10.1186/1471-2148-8-116)
Supplement: Additional File 1 — Supplementary Information. Pseudo-code for HOSC and details for the phages listed in Figure 6. [file 1471-2148-8-116-S1.doc]

Supplementary Information For

**Genome classification by gene distribution: an overlapping subspace clustering approach**

Jason Li^1^, Saman K. Halgamuge^1^, Sen-Lin Tang^2§^

^1^ Bioinformatics Section, Biomechanical Engineering, Department of Mechanical Engineering, the University of Melbourne, Australia

^2^ Research Center for Biodiversity, Academia Sinica, Taiwan

^§^Corresponding author

Email addresses:

JL: [j.li5@pgrad.unimelb.edu.au](mailto:lij@mame.mu.oz.au)

SKH: saman@unimelb.edu.au

ST: sltang@gate.sinica.edu.tw

Contents

[Pseudo code for O-HARP 2](#__RefHeading___Toc192655505)

[Details of the phages listed in Figure 6 7](#__RefHeading___Toc192655506)

# Pseudo code for O-HARP

##############################

#

# global variables

#

##############################

AllClusters – holds information about all the clusters at the current time point – these clusters maybe dismissed or merged later on

ScoreCache – stores a list of potential matches; each entry has information about two matching clusters, their merge score and associated subspace

t – clustering threshold

f – filtering threshold

############################################################

#

# buildScoreCache - called by main routine

#

############################################################

procedure buildScoreCache(NoOfDim)

empty the ScoreCache

loop over all combinations of cluster pairs among AllClusters

{

IF the pair has an ancestor-descendant relationship OR has been matched previously THEN

do not match this pair

ELSE

evaluateClusterPair(current pair, NoOfDim)

}

endprocedure

###############################################################

#

# evaluateClusterPair - called by buildScoreCache and updateScoreCache

#

###############################################################

procedure evaluateClusterPair(Pair, NoOfDim)

compute the Pair's merge score using Equation (1)

Subspace = subspace associated with the merge (the set of dimensions with R* > t during merge score computation - see EQ 1)

IF the number of dimensions of Subspace is equal to NoOfDim THEN

{

Obtain a list of data points associated with the existing clusters in Subspace

Compare this list against the data points of each of the two merging clusters

IF either cluster contains data intersecting this list AND has the original subspace different to the target Subspace THEN

do not match this pair so as to assert disjoint-ness in Subspace

ELSE

add this pair to ScoreCache

}

endprocedure

############################################################

#

# findBestMatch - called by main routine

#

############################################################

procedure findBestMatch()

initialize BestMatch as the score cache entry with the highest merge score

loop over all score cache entries that have the same subspace as BestMatch

{

IF the current entry is a descendant of BestMatch THEN

BestMatch = current entry

}

return BestMatch

endprocedure

############################################################

#

# updateParentChildRelationship - called by main routine

#

############################################################

procedure updateParentChildRelationship(NewCluster, BestPair, NoOfDim)

C1 = BestPair.Cluster1

C2 = BestPair.Cluster2

IF the number of dimensions of C1's subspace is larger than NoOfDim THEN

{

make C1 as NewCluster's direct parent

make NewCluster as a child of C1

}

ELSE

{

transfer C1's direct parents to NewCluster

replace C1 with NewCluster as a direct child to those parent clusters

remove cluster C1

}

repeat the above IF-THEN-ELSE statement for cluster C2

endprocedure

############################################################

#

# updateScoreCache - called by main routine

#

############################################################

procedure updateScoreCache(NewCluster, BestPair, NoOfDim)

initialize EntriesToRemove as an empty list

initialize ClustersToRecompute as an empty list

loop over all score cache entries except BestPair in ScoreCache

{

let C1 and C2 be the two clusters of the current score cache entry

IF C1 is either cluster of BestPair THEN

add C1 to ClustersToRecompute

IF C2 is either cluster of BestPair THEN

add C2 to ClustersToRecompute

IF the current entry's subspace is the same as the BestPair's subspace

AND IF C1 or C2 share one or more data points with the NewCluster THEN

add the current score cache entry to EntriesToRemove

IF either cluster of BestPair has been removed during match

AND IF the removed cluster happens to be C1 or C2 THEN

add the current score cache entry to EntriesToRemove

}

remove all entries in EntriesToRemove from ScoreCache

loop over all clusters in ClustersToRecompute

{

CandidatePair = (current cluster, NewCluster)

evaluateClusterPair(CandidatePair, NoOfDim)

}

endprocedure

############################################################

#

# filterOutInsignificantClusters - called by main routine

#

############################################################

procedure filterOutInsignificantClusters()

initialize CandidateSignificantClusters as an empty list

loop over all clusters

{

IF the current cluster has more than one data point AND satisfies Equation (5) THEN

add C to CandidateSignificantClusters

ELSE

mark C as insignificant

}

WHILE CandidateSignificantClusters is not empty

{

identify the cluster with the highest Score, call it the BestCluster

remove BestCluster from CandidateSignificantClusters

# the followingn two lines will recursively go through BestCluster's parents and

# childrens to decide their relative significance:

decideNextLevelSignificance(1, BestCluster, BestCluster)

decideNextLevelSignificance(0, BestCluster, BestCluster)

}

endprocedure

#########################################################################

#

# decideNextLevelSignificance - a recursive function used by filterOutInsignificantClusters

#

#########################################################################

procedure decideNextLevelSignificance(Direction, CurrentCluster, ClosestSignificantCluster)

{

IF Direction == 1 THEN

NextLevelClusters = CurrentCluster's direct parents

ELSE

NextLevelClusters = CurrentCluster's direct children

loop over all clusters in NextLevelClusters

{

SimiarityIndex = compute SI(current cluster, ClosestSignificantCluster) using

Equation (7)

NextClosestCluster = ClosestSignificantCluster

IF the current cluster is one of the CandidateSignificantClusters

AND IF SimiarityIndex > f THEN

{

mark the current cluster as significant

NextClosestCluster = current cluster

remove the current cluster from CandidateSignificantClusters

}

decideNextLevelSignificance(Direction, current cluster, NextClosest)

}

}

endprocedure

############################################################

#

# the main routine

#

############################################################

main routine:

loop over the number of dimensions in descending order (from max. number of dimensions to 1)

{

d = current number of dimension

buildScoreCache(d,t)

WHILE ScoreCache is not empty

{

BestPair = findBestMatch()

obtain the union set of data points between the two clusters of BestPair

create a new cluster with the union set, and associate it with the subspace

defined in BestPair

updateParentChildRelationship(the new cluster, BestPair, d)

updateScoreCache(the new cluster, BestPair, d)

}

}

filterOutInsignificantClusters()

end

# Details of the phages listed in Figure 6

Table S-1: Example positions of the five core genes depicted in Figure 6.

| **Group** | **Example Phage** | **Gene position (start-end)** | | | | |
| --- | --- | --- | --- | --- | --- | --- |
|  |  | **Integrase** | **Terminase large subunit** | **Portal** | **Holin** | **Lysin** |
| G1 | TP901-1 | 30-1487 | 13965-15353 | 15354-16712 | 33175-33441 | 33438-34727 |
| G2 | HP2 | 699-1712 | 10665-12488 | 9638-10675 | 19005-19241 | 19255-19794 |
| G3 | 187 | 21897-22943 | 478-1818 | 1830-3275 |  |  |
| G4 | HK97 | 21318-22388 | 542-2056 | 2056-3330 | 36248-36568 | 36552-37028 |
| G5 | Sfi21 | 24595-25674 | 549-2420 | 2621-3775 | 20996-21421 | 21637-22503 |
| G6 | P2 | 24462-25475 | 1221-2993 | 187-1221 | 6716-6997 | 6997-7494 |

## Group G1

Table S-2: Phage details for G1.

| **Phage** | **Refseq accession** | **Topology** | **Family** | **Host (genus)** | **Genome size (nt)** |
| --- | --- | --- | --- | --- | --- |
| bIL285 | [NC_002666](http://www.ncbi.nlm.nih.gov/entrez/viewer.fcgi?db=nucleotide&val=NC_002666) | linear | Siphoviridae | Lactococcus | 35538 |
| bIL286 | [NC_002667](http://www.ncbi.nlm.nih.gov/entrez/viewer.fcgi?db=nucleotide&val=NC_002667) | linear | Siphoviridae | Lactococcus | 41834 |
| bIL309 | [NC_002668](http://www.ncbi.nlm.nih.gov/entrez/viewer.fcgi?db=nucleotide&val=NC_002668) | linear | Siphoviridae | Lactococcus | 36949 |
| Tuc2009 | [NC_002703](http://www.ncbi.nlm.nih.gov/entrez/viewer.fcgi?db=nucleotide&val=NC_002703) | circular | Siphoviridae | Lactococcus | 38347 |
| phiLC3 | [NC_005822](http://www.ncbi.nlm.nih.gov/entrez/viewer.fcgi?db=nucleotide&val=NC_005822) | circular | Siphoviridae | Lactococcus | 32172 |
| phiETA | [NC_003288](http://www.ncbi.nlm.nih.gov/entrez/viewer.fcgi?db=nucleotide&val=NC_003288) | circular | Siphoviridae | Staphylococcus | 43081 |
| phBC6A52 | [NC_004821](http://www.ncbi.nlm.nih.gov/entrez/viewer.fcgi?db=nucleotide&val=NC_004821) | linear | Unclassified | Bacillus | 38472 |
| mu1/6 | [NC_007967](http://www.ncbi.nlm.nih.gov/entrez/viewer.fcgi?db=nucleotide&val=NC_007967) | linear | Siphoviridae | Streptomyces | 38194 |
| EJ-1 | [NC_005294](http://www.ncbi.nlm.nih.gov/entrez/viewer.fcgi?db=nucleotide&val=NC_005294) | linear | Myoviridae | Streptococcus | 42935 |
| BCJA1c | [NC_006557](http://www.ncbi.nlm.nih.gov/entrez/viewer.fcgi?db=nucleotide&val=NC_006557) | linear | Siphoviridae | Bacillus | 41092 |
| ul36 | [NC_004066](http://www.ncbi.nlm.nih.gov/entrez/viewer.fcgi?db=nucleotide&val=NC_004066) | linear | Siphoviridae | Lactococcus | 36798 |
| Lj928 | [NC_005354](http://www.ncbi.nlm.nih.gov/entrez/viewer.fcgi?db=nucleotide&val=NC_005354) | linear | Siphoviridae | Lactobacillus | 38384 |
| Lj965 | [NC_005355](http://www.ncbi.nlm.nih.gov/entrez/viewer.fcgi?db=nucleotide&val=NC_005355) | linear | Siphoviridae | Lactobacillus | 40190 |
| TP901-1 | [NC_002747](http://www.ncbi.nlm.nih.gov/entrez/viewer.fcgi?db=nucleotide&val=NC_002747) | linear | Siphoviridae | Lactococcus | 37667 |
| KC5a | [NC_007924](http://www.ncbi.nlm.nih.gov/entrez/viewer.fcgi?db=nucleotide&val=NC_007924) | circular | Siphoviridae | Lactobacillus | 38239 |
| phiadh | [NC_000896](http://www.ncbi.nlm.nih.gov/entrez/viewer.fcgi?db=nucleotide&val=NC_000896) | circular | Siphoviridae | Lactobacillus | 43785 |
| tp310-1 | [NC_009761](http://www.ncbi.nlm.nih.gov/entrez/viewer.fcgi?db=nucleotide&val=NC_009761) | linear | Unclassified | Staphylococcus | 41407 |
| 315.1 | [NC_004584](http://www.ncbi.nlm.nih.gov/entrez/viewer.fcgi?db=nucleotide&val=NC_004584) | linear | Unclassified | Streptococcus | 39538 |
| phiNM3 | [NC_008617](http://www.ncbi.nlm.nih.gov/entrez/viewer.fcgi?db=nucleotide&val=NC_008617) | linear | Siphoviridae | Staphylococcus | 44061 |
| phi 12 | [NC_004616](http://www.ncbi.nlm.nih.gov/entrez/viewer.fcgi?db=nucleotide&val=NC_004616) | linear | Siphoviridae | Staphylococcus | 44970 |
| phi 13 | [NC_004617](http://www.ncbi.nlm.nih.gov/entrez/viewer.fcgi?db=nucleotide&val=NC_004617) | linear | Siphoviridae | Staphylococcus | 42722 |
| phiPV83 | [NC_002486](http://www.ncbi.nlm.nih.gov/entrez/viewer.fcgi?db=nucleotide&val=NC_002486) | linear | Siphoviridae | Staphylococcus | 45636 |
| phiSLT | [NC_002661](http://www.ncbi.nlm.nih.gov/entrez/viewer.fcgi?db=nucleotide&val=NC_002661) | linear | Siphoviridae | Staphylococcus | 42942 |
| phiN315 | [NC_004740](http://www.ncbi.nlm.nih.gov/entrez/viewer.fcgi?db=nucleotide&val=NC_004740) | linear | Siphoviridae | Staphylococcus | 44082 |
| phiETA3 | [NC_008799](http://www.ncbi.nlm.nih.gov/entrez/viewer.fcgi?db=nucleotide&val=NC_008799) | circular | Siphoviridae | Staphylococcus | 43282 |
| phiPVL108 | [NC_008689](http://www.ncbi.nlm.nih.gov/entrez/viewer.fcgi?db=nucleotide&val=NC_008689) | linear | Siphoviridae | Staphylococcus | 44857 |
| tp310-1 | [NC_009761](http://www.ncbi.nlm.nih.gov/entrez/viewer.fcgi?db=nucleotide&val=NC_009761) | linear | Unclassified | Staphylococcus | 41407 |
| tp310-2 | [NC_009762](http://www.ncbi.nlm.nih.gov/entrez/viewer.fcgi?db=nucleotide&val=NC_009762) | linear | Unclassified | Staphylococcus | 45710 |
| phiNM | [NC_008583](http://www.ncbi.nlm.nih.gov/entrez/viewer.fcgi?db=nucleotide&val=NC_008583) | linear | Siphoviridae | Staphylococcus | 43128 |
| O1205 | [NC_004303](http://www.ncbi.nlm.nih.gov/entrez/viewer.fcgi?db=nucleotide&val=NC_004303) | linear | Siphoviridae | Streptococcus | 43075 |
| phi3396 | [NC_009018](http://www.ncbi.nlm.nih.gov/entrez/viewer.fcgi?db=nucleotide&val=NC_009018) | linear | Siphoviridae | Streptococcus | 38528 |
| MM1 | [NC_003050](http://www.ncbi.nlm.nih.gov/entrez/viewer.fcgi?db=nucleotide&val=NC_003050) | linear | Siphoviridae | Streptococcus | 40248 |
| P9 | [NC_009819](http://www.ncbi.nlm.nih.gov/entrez/viewer.fcgi?db=nucleotide&val=NC_009819) | linear | Unclassified | Streptococcus | 40539 |

## Group G2

Table S-3: Phage details for G2.

| **Phage** | **Refseq accession** | **Topology** | **Family** | **Host (genus)** | **Genome size (nt)** |
| --- | --- | --- | --- | --- | --- |
| HP1 | [NC_001697](http://www.ncbi.nlm.nih.gov/entrez/viewer.fcgi?db=nucleotide&val=NC_001697) | linear | Myoviridae | Haemophilus | 32355 |
| HP2 | [NC_003315](http://www.ncbi.nlm.nih.gov/entrez/viewer.fcgi?db=nucleotide&val=NC_003315) | linear | Myoviridae | Haemophilus | 31508 |
| phiO18P | [NC_009542](http://www.ncbi.nlm.nih.gov/entrez/viewer.fcgi?db=nucleotide&val=NC_009542) | linear | Myoviridae | Aeromonas | 33985 |
| K139 | [NC_003313](http://www.ncbi.nlm.nih.gov/entrez/viewer.fcgi?db=nucleotide&val=NC_003313) | linear | Myoviridae | Vibrio | 33106 |
| F108 | [NC_008193](http://www.ncbi.nlm.nih.gov/entrez/viewer.fcgi?db=nucleotide&val=NC_008193) | linear | Myoviridae | Pasteurella | 30505 |

## Group G3

Table S-4: Phage details for G3.

| **Phage** | **Refseq accession** | **Topology** | **Family** | **Host (genus)** | **Genome size (nt)** |
| --- | --- | --- | --- | --- | --- |
| EW | [NC_007056](http://www.ncbi.nlm.nih.gov/entrez/viewer.fcgi?db=nucleotide&val=NC_007056) | linear | Siphoviridae | Staphylococcus | 45286 |
| 187 | [NC_007047](http://www.ncbi.nlm.nih.gov/entrez/viewer.fcgi?db=nucleotide&val=NC_007047) | linear | Siphoviridae | Staphylococcus | 39620 |
| 37 | [NC_007055](http://www.ncbi.nlm.nih.gov/entrez/viewer.fcgi?db=nucleotide&val=NC_007055) | linear | Siphoviridae | Staphylococcus | 43681 |
| 42e | [NC_007052](http://www.ncbi.nlm.nih.gov/entrez/viewer.fcgi?db=nucleotide&val=NC_007052) | linear | Siphoviridae | Staphylococcus | 45861 |
| 69 | [NC_007048](http://www.ncbi.nlm.nih.gov/entrez/viewer.fcgi?db=nucleotide&val=NC_007048) | linear | Siphoviridae | Staphylococcus | 42732 |
| 88 | [NC_007063](http://www.ncbi.nlm.nih.gov/entrez/viewer.fcgi?db=nucleotide&val=NC_007063) | linear | Siphoviridae | Staphylococcus | 43231 |
| P22 | [NC_002371](http://www.ncbi.nlm.nih.gov/entrez/viewer.fcgi?db=nucleotide&val=NC_002371) | circular | Podoviridae | Salmonella | 41724 |
| Tweety | [NC_009820](http://www.ncbi.nlm.nih.gov/entrez/viewer.fcgi?db=nucleotide&val=NC_009820) | linear | Siphoviridae | Mycobacterium | 58692 |
| Cjw1 | [NC_004681](http://www.ncbi.nlm.nih.gov/entrez/viewer.fcgi?db=nucleotide&val=NC_004681) | linear | Siphoviridae | Mycobacterium | 75931 |
| Omega | [NC_004688](http://www.ncbi.nlm.nih.gov/entrez/viewer.fcgi?db=nucleotide&val=NC_004688) | linear | Siphoviridae | Mycobacterium | 110865 |
| Che8 | [NC_004680](http://www.ncbi.nlm.nih.gov/entrez/viewer.fcgi?db=nucleotide&val=NC_004680) | linear | Siphoviridae | Mycobacterium | 59471 |
| Llij | [NC_008196](http://www.ncbi.nlm.nih.gov/entrez/viewer.fcgi?db=nucleotide&val=NC_008196) | linear | Siphoviridae | Mycobacterium | 56852 |
| PMC | [NC_008205](http://www.ncbi.nlm.nih.gov/entrez/viewer.fcgi?db=nucleotide&val=NC_008205) | linear | Siphoviridae | Mycobacterium | 56692 |
| D3 | [NC_002484](http://www.ncbi.nlm.nih.gov/entrez/viewer.fcgi?db=nucleotide&val=NC_002484) | linear | Siphoviridae | Pseudomonas | 56425 |
| F10 | [NC_007805](http://www.ncbi.nlm.nih.gov/entrez/viewer.fcgi?db=nucleotide&val=NC_007805) | linear | Siphoviridae | Pseudomonas | 39199 |
| ES18 | [NC_006949](http://www.ncbi.nlm.nih.gov/entrez/viewer.fcgi?db=nucleotide&val=NC_006949) | linear | Siphoviridae | Salmonella | 46900 |
| 2638A | [NC_007051](http://www.ncbi.nlm.nih.gov/entrez/viewer.fcgi?db=nucleotide&val=NC_007051) | linear | Siphoviridae | Staphylococcus | 41318 |

## Group G4

Table S-5: Phage details for G4.

| **Phage** | **Refseq accession** | **Topology** | **Family** | **Host (genus)** | **Genome size (nt)** |
| --- | --- | --- | --- | --- | --- |
| lambda | [NC_001416](http://www.ncbi.nlm.nih.gov/entrez/viewer.fcgi?db=nucleotide&val=NC_001416) | linear | Siphoviridae | Escherichia | 48502 |
| HK022 | [NC_002166](http://www.ncbi.nlm.nih.gov/entrez/viewer.fcgi?db=nucleotide&val=NC_002166) | linear | Siphoviridae | Escherichia | 40751 |
| HK97 | [NC_002167](http://www.ncbi.nlm.nih.gov/entrez/viewer.fcgi?db=nucleotide&val=NC_002167) | linear | Siphoviridae | Escherichia | 39732 |
| P22 | [NC_002371](http://www.ncbi.nlm.nih.gov/entrez/viewer.fcgi?db=nucleotide&val=NC_002371) | circular | Podoviridae | Salmonella | 41724 |
| Sf6 | [NC_005344](http://www.ncbi.nlm.nih.gov/entrez/viewer.fcgi?db=nucleotide&val=NC_005344) | circular | Podoviridae | Shigella | 39043 |
| D3 | [NC_002484](http://www.ncbi.nlm.nih.gov/entrez/viewer.fcgi?db=nucleotide&val=NC_002484) | linear | Siphoviridae | Pseudomonas | 56425 |
| phi644-2 | [NC_009235](http://www.ncbi.nlm.nih.gov/entrez/viewer.fcgi?db=nucleotide&val=NC_009235) | linear | Siphoviridae | Burkholderia | 48674 |
| ES18 | [NC_006949](http://www.ncbi.nlm.nih.gov/entrez/viewer.fcgi?db=nucleotide&val=NC_006949) | linear | Siphoviridae | Salmonella | 46900 |
| ST64B | [NC_004313](http://www.ncbi.nlm.nih.gov/entrez/viewer.fcgi?db=nucleotide&val=NC_004313) | circular | Unclassified | Salmonella | 40149 |

## Group G5

Table S-6: Phage details for G5.

| **Phage** | **Refseq accession** | **Topology** | **Family** | **Host (genus)** | **Genome size (nt)** |
| --- | --- | --- | --- | --- | --- |
| A118 | [NC_003216](http://www.ncbi.nlm.nih.gov/entrez/viewer.fcgi?db=nucleotide&val=NC_003216) | linear | Siphoviridae | Listeria | 40834 |
| phi1026b | [NC_005284](http://www.ncbi.nlm.nih.gov/entrez/viewer.fcgi?db=nucleotide&val=NC_005284) | linear | Siphoviridae | Burkholderia | 54865 |
| phi3626 | [NC_003524](http://www.ncbi.nlm.nih.gov/entrez/viewer.fcgi?db=nucleotide&val=NC_003524) | linear | Siphoviridae | Clostridium | 33507 |
| phiE125 | [NC_003309](http://www.ncbi.nlm.nih.gov/entrez/viewer.fcgi?db=nucleotide&val=NC_003309) | linear | Siphoviridae | Burkholderia | 53373 |
| phiAT3 | [NC_005893](http://www.ncbi.nlm.nih.gov/entrez/viewer.fcgi?db=nucleotide&val=NC_005893) | circular | Siphoviridae | Lactobacillus | 39166 |
| phiC2 | [NC_009231](http://www.ncbi.nlm.nih.gov/entrez/viewer.fcgi?db=nucleotide&val=NC_009231) | linear | Unclassified | Clostridium | 56538 |
| phi CD119 | [NC_007917](http://www.ncbi.nlm.nih.gov/entrez/viewer.fcgi?db=nucleotide&val=NC_007917) | linear | Myoviridae | Clostridium | 53325 |
| GBSV1 | [NC_008376](http://www.ncbi.nlm.nih.gov/entrez/viewer.fcgi?db=nucleotide&val=NC_008376) | linear | Unclassified | Geobacillus | 34683 |
| A2 | [NC_004112](http://www.ncbi.nlm.nih.gov/entrez/viewer.fcgi?db=nucleotide&val=NC_004112) | linear | Siphoviridae | Lactobacillus | 43411 |
| BK5-T | [NC_002796](http://www.ncbi.nlm.nih.gov/entrez/viewer.fcgi?db=nucleotide&val=NC_002796) | circular | Siphoviridae | Lactococcus | 40003 |
| PSA | [NC_003291](http://www.ncbi.nlm.nih.gov/entrez/viewer.fcgi?db=nucleotide&val=NC_003291) | linear | Siphoviridae | Listeria | 37618 |
| PVL | [NC_002321](http://www.ncbi.nlm.nih.gov/entrez/viewer.fcgi?db=nucleotide&val=NC_002321) | linear | Siphoviridae | Staphylococcus | 41401 |
| EW | [NC_007056](http://www.ncbi.nlm.nih.gov/entrez/viewer.fcgi?db=nucleotide&val=NC_007056) | linear | Siphoviridae | Staphylococcus | 45286 |
| 71 | [NC_007059](http://www.ncbi.nlm.nih.gov/entrez/viewer.fcgi?db=nucleotide&val=NC_007059) | linear | Siphoviridae | Staphylococcus | 43114 |
| 55 | [NC_007060](http://www.ncbi.nlm.nih.gov/entrez/viewer.fcgi?db=nucleotide&val=NC_007060) | linear | Siphoviridae | Staphylococcus | 41902 |
| Sfi21 | [NC_000872](http://www.ncbi.nlm.nih.gov/entrez/viewer.fcgi?db=nucleotide&val=NC_000872) | linear | Siphoviridae | Streptococcus | 40739 |
| RTP | [NC_007603](http://www.ncbi.nlm.nih.gov/entrez/viewer.fcgi?db=nucleotide&val=NC_007603) | linear | Siphoviridae | Escherichia | 46219 |
| phi105 | [NC_004167](http://www.ncbi.nlm.nih.gov/entrez/viewer.fcgi?db=nucleotide&val=NC_004167) | circular | Siphoviridae | Bacillus | 39325 |
| bIL170 | [NC_001909](http://www.ncbi.nlm.nih.gov/entrez/viewer.fcgi?db=nucleotide&val=NC_001909) | linear | Siphoviridae | Lactococcus | 31754 |
| 712 | [NC_008370](http://www.ncbi.nlm.nih.gov/entrez/viewer.fcgi?db=nucleotide&val=NC_008370) | circular | Siphoviridae | Lactococcus | 30510 |
| P008 | [NC_008363](http://www.ncbi.nlm.nih.gov/entrez/viewer.fcgi?db=nucleotide&val=NC_008363) | linear | Siphoviridae | Lactococcus | 28538 |
| jj50 | [NC_008371](http://www.ncbi.nlm.nih.gov/entrez/viewer.fcgi?db=nucleotide&val=NC_008371) | circular | Siphoviridae | Lactococcus | 27453 |
| 7201 | [NC_002185](http://www.ncbi.nlm.nih.gov/entrez/viewer.fcgi?db=nucleotide&val=NC_002185) | linear | Siphoviridae | Streptococcus | 35466 |
| 47 | [NC_007054](http://www.ncbi.nlm.nih.gov/entrez/viewer.fcgi?db=nucleotide&val=NC_007054) | linear | Siphoviridae | Staphylococcus | 44777 |
| CNPH82 | [NC_008722](http://www.ncbi.nlm.nih.gov/entrez/viewer.fcgi?db=nucleotide&val=NC_008722) | linear | Siphoviridae | Staphylococcus | 43420 |
| 77 | [NC_005356](http://www.ncbi.nlm.nih.gov/entrez/viewer.fcgi?db=nucleotide&val=NC_005356) | linear | Siphoviridae | Staphylococcus | 41708 |
| BFK20 | [NC_009799](http://www.ncbi.nlm.nih.gov/entrez/viewer.fcgi?db=nucleotide&val=NC_009799) | linear | Siphoviridae | Corynebacterium | 42969 |
| Sfi11 | [NC_002214](http://www.ncbi.nlm.nih.gov/entrez/viewer.fcgi?db=nucleotide&val=NC_002214) | linear | Siphoviridae | Streptococcus | 39807 |
| WBeta | [NC_007734](http://www.ncbi.nlm.nih.gov/entrez/viewer.fcgi?db=nucleotide&val=NC_007734) | linear | Siphoviridae | Bacillus | 40867 |
| Fah | [NC_007814](http://www.ncbi.nlm.nih.gov/entrez/viewer.fcgi?db=nucleotide&val=NC_007814) | linear | Siphoviridae | Bacillus | 37974 |
| Gamma | [NC_007458](http://www.ncbi.nlm.nih.gov/entrez/viewer.fcgi?db=nucleotide&val=NC_007458) | linear | Siphoviridae | Bacillus | 37253 |
| Cherry | [NC_007457](http://www.ncbi.nlm.nih.gov/entrez/viewer.fcgi?db=nucleotide&val=NC_007457) | linear | Siphoviridae | Bacillus | 36615 |
| 80alpha | [NC_009526](http://www.ncbi.nlm.nih.gov/entrez/viewer.fcgi?db=nucleotide&val=NC_009526) | linear | Siphoviridae | Staphylococcus | 43864 |
| PH15 | [NC_008723](http://www.ncbi.nlm.nih.gov/entrez/viewer.fcgi?db=nucleotide&val=NC_008723) | linear | Siphoviridae | Staphylococcus | 44041 |
| Sfi19 | [NC_000871](http://www.ncbi.nlm.nih.gov/entrez/viewer.fcgi?db=nucleotide&val=NC_000871) | linear | Siphoviridae | Streptococcus | 37370 |
| DT1 | [NC_002072](http://www.ncbi.nlm.nih.gov/entrez/viewer.fcgi?db=nucleotide&val=NC_002072) | linear | Siphoviridae | Streptococcus | 34815 |
| Lc-Nu | [NC_007501](http://www.ncbi.nlm.nih.gov/entrez/viewer.fcgi?db=nucleotide&val=NC_007501) | linear | Siphoviridae | Lactobacillus | 36466 |

## Group G6

Table S-7: Phage details for G6.

| **Phage** | **Refseq accession** | **Topology** | **Family** | **Host (genus)** | **Genome size (nt)** |
| --- | --- | --- | --- | --- | --- |
| L-413C | [NC_004745](http://www.ncbi.nlm.nih.gov/entrez/viewer.fcgi?db=nucleotide&val=NC_004745) | linear | Myoviridae | Yersinia | 30728 |
| WPhi | [NC_005056](http://www.ncbi.nlm.nih.gov/entrez/viewer.fcgi?db=nucleotide&val=NC_005056) | linear | Myoviridae | Escherichia | 32684 |
| 186 | [NC_001317](http://www.ncbi.nlm.nih.gov/entrez/viewer.fcgi?db=nucleotide&val=NC_001317) | linear | Myoviridae | Escherichia | 30624 |
| P2 | [NC_001895](http://www.ncbi.nlm.nih.gov/entrez/viewer.fcgi?db=nucleotide&val=NC_001895) | linear | Myoviridae | Escherichia | 33593 |
| PsP3 | [NC_005340](http://www.ncbi.nlm.nih.gov/entrez/viewer.fcgi?db=nucleotide&val=NC_005340) | linear | Myoviridae | Salmonella | 30636 |
